# ChatGPT use and learner autonomy among EFL university students: the mediating role of English speaking anxiety

**DOI:** 10.3389/fpsyg.2026.1834791

**Published:** 2026-06-26

**Authors:** Amal Mubarak Alsuwaylim, Mohammed A. Alshehri, Ali Lamouchi, Mohamed Sayed Abdellatif, Ashraf Ragab Ibrahim, Mohamed Ali Nemt-allah

**Affiliations:** 1 Department of Psychology, College of Education, Prince Sattam Bin Abdulaziz University, Al-Kharj, Saudi Arabia; 2 Department of Curriculum and Instruction, College of Education, King Saud University, Riyadh, Saudi Arabia; 3 Department of English Language and Literature, College of Science and Humanities, Prince Sattam bin Abdulaziz University, Al-Kharj, Saudi Arabia; 4 Educational Psychology and Statistics Department, Faculty of Education, Al-Azhar University, Dakahlia, Egypt

**Keywords:** affective filter hypothesis, ChatGPT use, EFL university students, English speaking anxiety, learner autonomy, mediation analysis

## Abstract

Despite the rapid proliferation of ChatGPT in EFL educational contexts, to the best of current knowledge, no identified study has simultaneously modeled ChatGPT use, English speaking anxiety, and learner autonomy within a single structural framework. The present study aims to investigate the mediating role of English speaking anxiety in the relationship between ChatGPT use and learner autonomy among EFL university students. A quantitative, cross-sectional survey design is employed with a main sample of 852 EFL students drawn from three Egyptian public universities. Participants complete three validated instruments: the ChatGPT usage scale, the Foreign language classroom speaking anxiety scale, and the learner autonomy scale. Data are analyzed using Pearson correlation and Hayes's PROCESS macro (Model 4) with 5,000 bootstrap resamples. Results reveal that ChatGPT use is negatively associated with speaking anxiety and positively associated with learner autonomy, while speaking anxiety negatively predicts learner autonomy. Mediation analysis confirms that speaking anxiety partially mediates the ChatGPT use–learner autonomy relationship, accounting for 34.80% of the total effect. These findings are consistent with the integration of the Affective Filter Hypothesis and Self-Determination Theory as complementary frameworks for understanding AI-mediated language learning, though causal interpretation is limited by the cross-sectional design and the reliance on a classroom-based anxiety measure. The results tentatively suggest the potential value of anxiety-targeted ChatGPT integration strategies in EFL curricula, pending replication through longitudinal and experimentally controlled designs.

## 1 Introduction

The rapid proliferation of artificial intelligence (AI) has fundamentally transformed educational systems worldwide, shifting from experimental applications to deeply embedded pedagogical and administrative infrastructure (Chen et al., 2020; Tapalova and Zhiyenbayeva, 2022). AI-powered tools now support personalized learning pathways, intelligent tutoring systems, real-time feedback, and automated assessment across diverse disciplines (Almasri, 2024; Bahroun et al., 2023). The emergence of generative AI—particularly ChatGPT—has intensified scholarly interest, with bibliometric analyses documenting an exponential surge in AI-in-education research in 2023 (Bahroun et al., 2023; Yan et al., 2024). ChatGPT has since distinguished itself as the most widely adopted generative AI tool in educational practice (Adiguzel et al., 2023; Kasneci et al., 2023), gaining particular prominence in language learning contexts where it serves as a conversational partner, personalized tutor, and feedback provider for EFL learners (Liu et al., 2024; Qu and Wu, 2024; Xiao and Zhi, 2023).

EFL university learners occupy a particularly significant position in the study of AI-assisted language learning, given the compounding challenges they face within higher education settings. Students in these contexts grapple with persistent language barriers, academic literacy demands, and pervasive foreign language anxiety—factors that systematically undermine confidence and performance (Ross and Stuckler, 2025; Song and Song, 2023). Many learners study in environments offering limited access to authentic English input, large class sizes, and constrained teacher feedback, prompting widespread adoption of ChatGPT as an on-demand conversational partner and personalized tutor (Karatas et al., 2024; Liu et al., 2024; Xiao and Zhi, 2023). These structural deficits intersect with intense academic pressure, heightening anxiety and motivational challenges across writing, speaking, and vocabulary tasks (Alwasidi and Al-Khalifah, 2025; Panggua et al., 2025; Qu and Wu, 2024). Consequently, the EFL university context constitutes a critical site for examining both the affordances and risks of ChatGPT integration in language learning (Deep et al., 2025; Lo et al., 2024).

Learner autonomy—broadly defined as the capacity to take charge of one's own learning through goal-setting, strategy selection, self-monitoring, and outcome evaluation—has emerged as a cornerstone construct in contemporary EFL pedagogy and applied linguistics (Chong and Reinders, 2022; Tuan, 2021). It is widely regarded as the ultimate aim of learner-centered and communicative language curricula, positioning students as active agents rather than passive recipients of instruction (Alrashidi, 2022; Dang, 2025; Han, 2021). At the university level, empirical evidence consistently links higher autonomy to stronger L2 motivation, greater academic achievement, and sustained self-directed learning beyond the classroom (Gocić and Jankovic, 2022; Liu, 2015; Melvina and Julia, 2021; Sakrak-Ekin and Balçikanli, 2019). These documented benefits render learner autonomy an indispensable variable in investigating how emerging technologies such as ChatGPT shape EFL learners' independent language-learning behaviors (Nguyen and Hue, 2025; Wei, 2023).

A growing body of empirical evidence suggests that ChatGPT functions as an on-demand, personalized language tutor that meaningfully supports independent learning behaviors among university EFL learners. Students across diverse contexts report using ChatGPT for iterative writing feedback, reading strategy development, and self-initiated language practice—functions that directly scaffold goal-setting, self-monitoring, and reflective strategy use central to learner autonomy (Al Zakwani and Binu, 2025; Xiao and Zhi, 2023; Zhang et al., 2025). Experimental and mixed-methods research further demonstrates that ChatGPT-facilitated learning produces significantly greater autonomy gains than teacher-directed instruction, as learners progressively shift from dependence toward authentic self-regulation (Lee et al., 2024; Nguyen and Hue, 2025; Xu et al., 2024). Its 24/7 availability and personalized responsiveness expand informal, self-initiated learning opportunities beyond classroom boundaries (Du and Alm, 2024; Liu et al., 2024; Zakarneh et al., 2025). Nevertheless, scholars caution that uncritical adoption risks fostering over-reliance, warranting guided integration to sustain genuine autonomous development (Alruwaili and Kianfar, 2025; Lo et al., 2024; Slamet and Basthomi, 2025).

English speaking anxiety—a domain-specific manifestation of foreign language anxiety tied exclusively to oral communication—represents one of the most pervasive and debilitating psychological barriers confronting EFL university students (Hanifa, 2018; Ross and Stuckler, 2025; Thach and Khau, 2025). Arising from intertwined cognitive-linguistic sources such as limited vocabulary, fear of negative evaluation, and low perceived oral proficiency, alongside affective factors including shyness, self-inferiority, and communication apprehension, it functions as a powerful inhibitory force on spoken language performance (Malik et al., 2021, 2024; Suratin and Sribayak, 2025). Cognitively, it disrupts attention, impairs lexical retrieval, and operates as an affective filter that constrains both comprehension and production (Liu, 2018; Thach and Khau, 2025). Emotionally, it generates persistent tension, dread, and diminished enjoyment in speaking contexts (Bozkurt and Aydin, 2023; Zhao et al., 2025). Behaviorally, it manifests as avoidance, reduced participation, and measurably lower oral test performance, thereby mediating the relationship between learner characteristics and communicative outcomes (Liu, 2018; Malik et al., 2024; Ren et al., 2025).

Emerging theoretical and empirical work suggests that AI-mediated communication creates low-stakes interactional spaces that meaningfully attenuate English speaking anxiety among EFL learners. Unlike teacher- or peer-directed communication, interactions with AI tools such as ChatGPT eliminate social-evaluative threats, reduce face loss, and lower affective filter activation—conditions learners consistently identify as enabling safer, less inhibited oral practice (Bashori et al., 2020; Fauzi et al., 2025). Experimental and observational studies confirm that AI-assisted speaking practice significantly reduces anxiety while simultaneously enhancing self-efficacy, enjoyment, and willingness to communicate (Bashori et al., 2021; Wardat and Akour, 2025; Yan et al., 2025; Zhang and Liu, 2025). Critically, this anxiety reduction appears to initiate a facilitative chain: as learners grow more confident, they engage more persistently in self-initiated, autonomous practice beyond the classroom (Liu et al., 2024; Qiao and Zhao, 2023; Sayed et al., 2024; Wei, 2023). These converging findings position English speaking anxiety as a theoretically plausible mediator between ChatGPT use and the development of learner autonomy—a relationship warranting systematic empirical investigation (AlTwijri and Alghizzi, 2024; Crompton et al., 2024; Nhan et al., 2025).

Grounded in the Affective Filter Hypothesis and Self-Determination Theory (SDT), it is theoretically coherent to propose that English speaking anxiety mediates the relationship between ChatGPT use and learner autonomy. SDT (Deci and Ryan, 1985, 2000) identifies three basic psychological needs—autonomy, competence, and relatedness. The present model centers autonomy as the primary outcome given its direct operationalization in EFL research. Competence is theoretically implicated within the ChatGPT–anxiety pathway, as pressure-free AI practice restores perceived oral competence, thereby reducing anxiety. Relatedness is excluded as theoretically peripheral to dyadic human–AI interaction. From an SDT perspective, anxiety undermines learners' basic needs for competence and autonomy; as ChatGPT restores perceived competence through pressure-free practice, learners progressively adopt more self-determined behaviors (Alamer et al., 2022; Annamalai and Nasor, 2025; Du and Alm, 2024; Li and Chiu, 2025; Noels et al., 2000).

Krashen (1982) Affective Filter Hypothesis posits that anxiety operates as an inhibitory barrier to language production and acquisition. In the present model, speaking anxiety operationalizes this filter as a stable, classroom-situated dispositional tendency—capturing learners‘ chronic communicative inhibition in teacher- and peer-present contexts (Apple, 2013; Horwitz et al., 1986)—rather than anxiety experienced during AI interactions *per se*. ChatGPT use represents the filter-attenuating mechanism through which sustained low-stakes AI engagement may progressively reduce learners' dispositional classroom speaking anxiety (Fauzi et al., 2025; Wardat and Akour, 2025). Nevertheless, it should be explicitly acknowledged that the FLCSAS was designed for traditional classroom settings, and its application here assumes that anxiety reductions generalize from AI-mediated practice to classroom-based speaking experiences (Bashori et al., 2021).

Empirical evidence corroborates this sequential logic, demonstrating that anxiety reduction in AI-enhanced contexts directly precedes gains in self-regulation and learner autonomy (Al Zakwani and Binu, 2025; Ebadi et al., 2025; Nguyen and Hue, 2025; Sayed et al., 2024). The mediation pathway is theoretically predicted rather than inductively derived from prior findings: anxiety reduction is a necessary intermediate mechanism within both frameworks, and cited empirical studies serve as convergent evidence consistent with this theoretical logic rather than as its foundation.

Despite the growing body of literature on ChatGPT in EFL contexts, a critical empirical gap persists: to the best of our knowledge, no identified study has simultaneously modeled ChatGPT use, English speaking anxiety, and learner autonomy within a single structural framework. Existing research examines these constructs predominantly in isolated pairs—ChatGPT and autonomy (Al Zakwani and Binu, 2025; Nguyen and Hue, 2025; Sayed et al., 2024), or ChatGPT and affective outcomes (Almineeai et al., 2025; Budhathoki et al., 2024)—without formally testing speaking anxiety as a mediating variable. Furthermore, systematic reviews highlight the relative underrepresentation of speaking-focused and mediation-based designs in ChatGPT-for-EFL research (Lo et al., 2024; Zou et al., 2024). Theoretically, autonomy and speaking anxiety remain unintegrated within AI-mediated learning frameworks (Du and Alm, 2024; Xu et al., 2024; Elov et al., 2025).

The present study aims to investigate the mediating role of English speaking anxiety in the relationship between ChatGPT use and learner autonomy among EFL university students at three Egyptian public universities. Specifically, the study examines the direct effect of ChatGPT use on learner autonomy, the effect of ChatGPT use on English speaking anxiety, the effect of English speaking anxiety on learner autonomy, and the indirect effect of ChatGPT use on learner autonomy through the mediating role of English speaking anxiety. The study further seeks to determine whether speaking anxiety functions as a partial or full mediator within this pathway, thereby contributing empirical evidence to the theoretically proposed but previously untested sequential relationship among these three constructs within a single structural framework.

## 2 Method

### 2.1 Study design

This study employed a quantitative, cross-sectional survey design to examine the relationships among ChatGPT use, English speaking anxiety, and learner autonomy among EFL university students, with speaking anxiety tested as a mediating variable in the ChatGPT use–learner autonomy relationship.

### 2.2 Participants

The study involved two samples drawn from three Egyptian public universities: Al-Azhar University, Zagazig University, and Kafr El-Sheikh University. A psychometric sample (*N* = 718) was used for scale validation, with participants ranging in age from 18 to 25 years (*M* = 21.46, *SD* = 2.26). A main sample (*N* = 852) was subsequently recruited for hypothesis testing, with participants ranging in age from 18 to 24 years (*M* = 20.37, *SD* = 1.51). Full demographic characteristics of both samples are presented in Table 1.

**Table 1:** Demographic characteristics of the psychometric and main samples.

Variable	Category	Psychometric sample		Main sample	
		*N*	*%*	*N*	*%*
University	Al-Azhar	302	42.1	375	44.0
	Zagazig	211	29.4	171	20.1
	Kafr El-Sheikh	205	28.6	306	35.9
Gender	Male	272	37.9	296	34.7
	Female	446	62.1	556	65.3
Academic level	First year	170	23.7	186	21.8
	Second year	204	28.4	253	29.7
	Third year	186	25.9	255	29.9
	Fourth year	158	22.0	158	18.5
Language	English	400	55.7	493	57.9
	French	318	44.3	359	42.1
Department	English	275	38.3	264	31.0
	French	237	33.0	191	22.4
	Arts	206	28.7	397	46.6
GPA	Excellent	149	20.8	158	18.5
	Very Good	296	41.2	333	39.1
	Good	212	29.5	284	33.3
	Acceptable	61	8.5	77	9.0

*N*, frequency; %, valid percentage of total sample.

### 2.3 Measures

ChatGPT Usage Scale developed and validated by Nemt-Allah et al. (2024), comprising 15 items distributed across three subscales: Academic Writing Aid (7 items), Academic Task Support (4 items), and Reliance and Trust (4 items). Items are rated on a five-point Likert scale ranging from 1 (strongly disagree) to 5 (strongly agree). Confirmatory factor analysis (CFA) in the present psychometric sample yielded acceptable model fit: χ^2^/df = 1.063, Root Mean Square Error of Approximation (RMSEA) = 0.049, Goodness-of-Fit Index (GFI) = 0.983, Comparative Fit Index (CFI) = 0.998, and Tucker–Lewis Index (TLI) = 0.998. Reliability estimates were strong at the subscale level (ω ranging from 0.753 to 0.869) and at the total scale level (ω = 0.876, α = 0.877). Although the ChatGPT Usage Scale primarily captures writing and general academic support functions, its relevance to speaking anxiety is justified on two grounds. First, it is theoretically assumed that general ChatGPT engagement may foster a low-stakes interactional relationship with AI that could generalize to oral self-perception and communicative confidence, though whether participants' engagement was sufficiently habitual or sustained to produce such effects was not empirically assessed. Second, it is theoretically proposed—rather than empirically verified—that affective filter reduction may operate at the level of overall AI interaction comfort rather than modality-specific use.

The Foreign Language Classroom Speaking Anxiety Scale (FLCSAS) developed by Apple (2013). The scale consists of 20 items reflecting a unidimensional construct and uses a five-point Likert response format ranging from 1 (strongly disagree) to 5 (strongly agree). CFA in the psychometric sample indicated excellent fit: χ^2^/df = 0.875, RMSEA = 0.030, GFI = 0.980, CFI = 0.998, and TLI = 0.985. The scale demonstrated excellent internal consistency (ω = 0.944, α = 0.940).

The Learner Autonomy Scale developed by Sereti and Giossos (2018), consisting of 24 items distributed across four dimensions: special Ability to Self-Management (8 items), Special Psychological Disposition (6 items), General Ability to Self-Management (7 items), and General Psychological Disposition (3 items). Items are rated on a five-point Likert scale from 1 (totally disagree) to 5 (absolutely agree). CFA results in the psychometric sample supported adequate model fit: χ^2^/df = 0.952, RMSEA = 0.050, GFI = 0.973, CFI = 0.998, and TLI = 0.991. Reliability was strong across subscales (ω ranging from 0.742 to 0.900) and at the total scale level (ω = 0.917, α = 0.916).

### 2.4 Common method bias

To assess the risk of common method bias, Harman's single-factor test was conducted. A single factor accounted for 29.37% of the total variance, falling well below the 50% threshold, suggesting that common method variance does not pose a substantial threat to the validity of the findings.

### 2.5 Procedures

Data were collected via Google Forms over the period of September 3 to November 18, 2025, corresponding to the first academic semester of the 2025/2026 academic year. Given that participants are native Arabic speakers, all scales originally developed in English were translated into Arabic and then back-translated into English by a panel of three bilingual content experts to ensure linguistic equivalence and conceptual accuracy. The ChatGPT Usage Scale was exempt from this procedure, as it was originally developed in Arabic. Data were analyzed using SPSS version 27 and AMOS version 26.

### 2.6 Data analysis

Descriptive statistics and bivariate Pearson correlations were computed to characterize the study variables and examine their interrelationships. Mediation analysis was subsequently conducted using the PROCESS macro (Model 4) with 5,000 bootstrap resamples and 95% bias-corrected confidence intervals to test the indirect effect of ChatGPT use on learner autonomy through English speaking anxiety.

## 3 Results

### 3.1 Preliminary analyses

Prior to testing the hypothesized mediation model, preliminary analyses were conducted to characterize the study variables and evaluate the assumptions underlying subsequent inferential procedures. Descriptive statistics—including minimum and maximum scores, means, standard errors, skewness, and kurtosis—were computed for all subscales and composite scores across the three instruments. As displayed in Table 2, ChatGPT Use yielded a total mean of 48.73 (SE = 0.42), with subscale means of 22.74, 12.99, and 12.99 for Academic Writing Aid, Academic Task Support, and Reliance and Trust, respectively. Speaking Anxiety produced a mean of 64.98 (SE = 0.64). For Learner Autonomy, the total mean was 73.99 (SE = 0.36), with subscale means ranging from 8.75 for General Psychological Disposition to 25.99 for Special Ability to Self-Management. Inspection of skewness and kurtosis indices across all variables indicated values within acceptable limits (|skewness| < 1.0; |kurtosis| < 1.0), supporting the assumption of approximate univariate normality sufficient for the planned analyses.

**Table 2:** Descriptive statistics for all study variables.

Variable	Min	Max	Mean	*SE*	Skewness	Kurtosis
Academic Writing Aid (AWA)	7.00	35.00	22.74	0.24	−0.23	−0.84
Academic Task Support (ATS)	4.00	20.00	12.99	0.14	−0.23	−0.80
Reliance and Trust (RT)	4.00	20.00	12.99	0.14	−0.23	−0.70
ChatGPT use (total)	16.00	75.00	48.73	0.42	−0.22	−0.61
Speaking anxiety	21.00	100.00	64.98	0.64	−0.23	−0.79
Special ability–self-management	8.00	40.00	25.99	0.27	−0.25	−0.76
Special psychological disposition	6.00	30.00	16.51	0.20	0.21	−0.75
General ability–self-management	7.00	35.00	22.74	0.23	−0.22	−0.67
General psychological disposition	4.00	14.00	8.75	0.06	0.16	−0.43
Learner autonomy (Total)	47.00	104.00	73.99	0.36	−0.09	−0.51

*SE*, standard error of the mean. Skewness and kurtosis values within 1.0 indicate acceptable normality.

### 3.2 Bivariate correlations

Pearson correlation analysis was conducted to examine the pairwise associations among ChatGPT use, English speaking anxiety, and learner autonomy. The results, summarized in Table 3, revealed that ChatGPT use was significantly and negatively correlated with speaking anxiety (*r* = −0.364, *p* < 0.001), indicating that higher levels of ChatGPT engagement were associated with lower anxiety about speaking English in the classroom. ChatGPT use was also significantly and positively correlated with learner autonomy (*r* = 0.344, *p* < 0.001), suggesting that greater use of the tool corresponded with higher levels of autonomous learning behavior. Furthermore, speaking anxiety exhibited a significant negative association with learner autonomy (*r* = −0.411, *p* < 0.001), such that learners reporting higher anxiety levels demonstrated comparatively lower autonomy. Taken together, these bivariate relationships provide preliminary support for the proposed mediation pathway and are theoretically consistent with the role of anxiety as an intermediary variable linking ChatGPT use to learner autonomy.

**Table 3:** Pearson correlation matrix among study variables.

Variable	1	2	3
1. ChatGPT use	1		
2. Speaking anxiety	−0.364^**^	1	
3. Learner autonomy	0.344^**^	−0.411^**^	1

^**^p < 0.01 (two-tailed).

### 3.3 Mediation analysis: path coefficients

To test the hypothesized mediation model, Hayes's PROCESS macro (Model 4) was applied with 5,000 bootstrap resamples and 95% bias-corrected confidence intervals (*N* = 852). The analysis simultaneously estimated three paths: the effect of ChatGPT use (X) on speaking anxiety (M), the effect of speaking anxiety (M) on learner autonomy (Y), and the direct effect of ChatGPT use (X) on learner autonomy (Y) after controlling for the mediator. Prior to mediation analysis, multicollinearity diagnostics were conducted. Variance inflation factors (VIF) for all predictors fell below the recommended threshold of 3.0 (ChatGPT Use: VIF = 1.154; Speaking Anxiety: VIF = 1.154), confirming the absence of multicollinearity concerns in the regression-based mediation model. The individual path coefficients are presented in Table 4.

**Table 4:** Path coefficients in the mediation model.

Outcome variable	Predictor variable	β (std.)	*B* (unstd.)	*SE*	*T*	*p*	95% CI
Learner autonomy (Y)	ChatGPT use (X)	0.2246	0.193	0.028	6.87	0.000	[0.13, 0.24]
Learner autonomy (Y)	Speaking anxiety (M)	−0.3293	−0.187	0.018	−10.07	0.000	[−0.22, −0.15]
Speaking anxiety (M)	ChatGPT use (X)	−0.3637	−0.547	0.048	−11.38	0.000	[−0.64, −0.45]

β, standardized coefficient; *B*, unstandardized coefficient; *SE*, standard error; CI, confidence interval computed via 5,000 bootstrap resamples. All paths significant at *p* < 0.001.

Results indicated that ChatGPT use significantly predicted speaking anxiety [β = −0.364, *B* = −0.548, *SE* = 0.048, *t* = −11.38, *p* < 0.001, 95% CI (−0.64, −0.45)], confirming that higher ChatGPT engagement was associated with meaningfully lower levels of English speaking anxiety. In turn, speaking anxiety was a significant negative predictor of learner autonomy [β = −0.329, *B* = −0.188, *SE* = 0.019, *t* = −10.07, *p* < 0.001, 95% CI (−0.22, −0.15)], demonstrating that greater anxiety suppressed autonomous learning behavior. ChatGPT use also exerted a significant direct positive effect on learner autonomy even after controlling for speaking anxiety [β = 0.225, *B* = 0.193, *SE* = 0.028, *t* = 6.87, *p* < 0.001, 95% CI (0.13, 0.24)]. The mediation model demonstrated adequate explanatory power, accounting for 13.23% of the variance in speaking anxiety (*R*^2^ = 0.132) and 21.27% of the variance in learner autonomy (*R*^2^ = 0.213), both of which are considered meaningful effect sizes within social-behavioral mediation research (Cohen, 1988).

### 3.4 Mediation analysis: total, direct, and indirect effects

**Figure 1:**
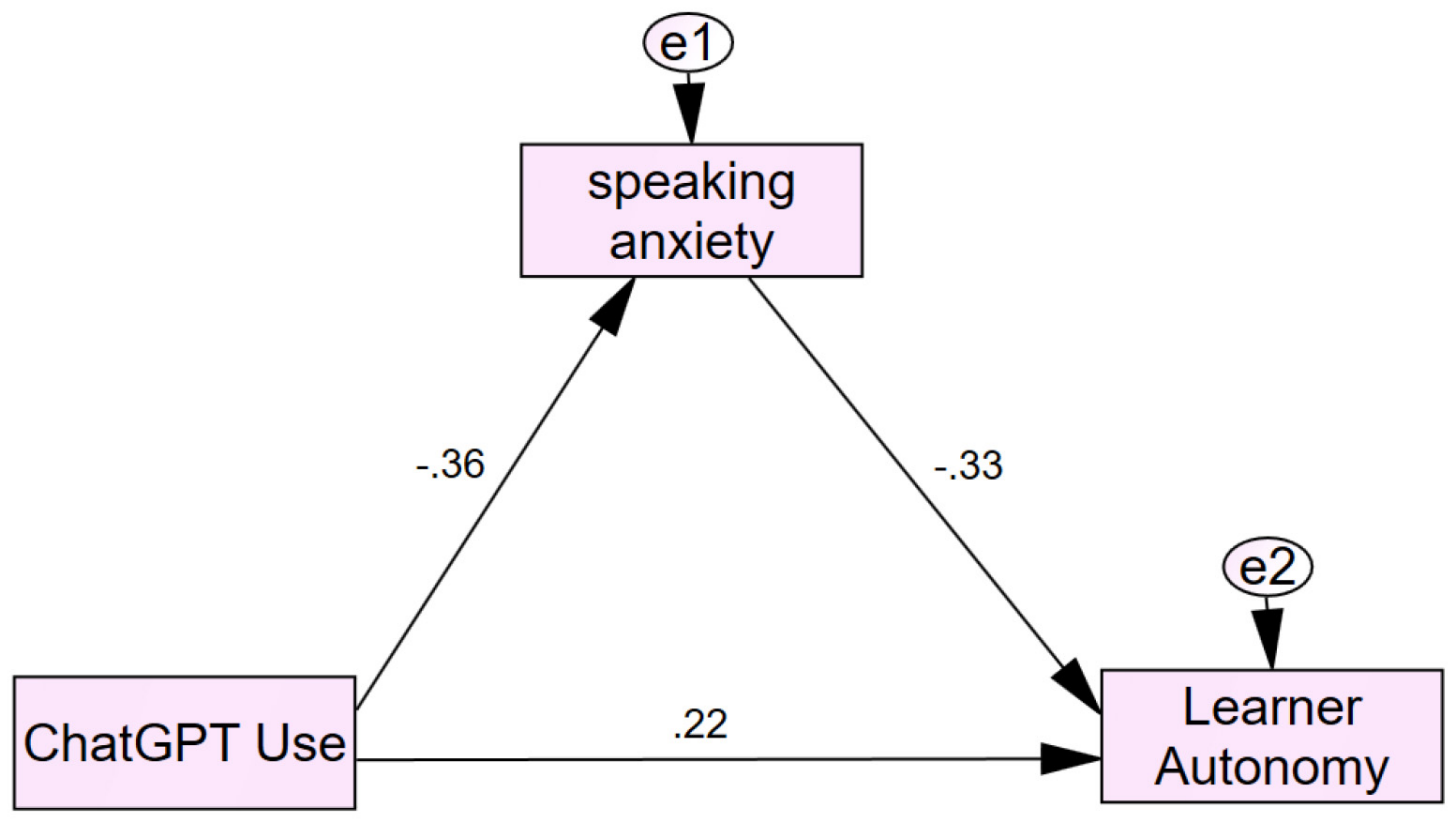
Mediation model of ChatGPT use, speaking anxiety, and learner autonomy.

The decomposition of effects is reported in Table 5. The total effect of ChatGPT use on learner autonomy was positive and statistically significant [*B* = 0.296, β = 0.344, *SE* = 0.028, 95% CI (0.24, 0.35)]. Following the introduction of speaking anxiety as a mediator, the direct effect of ChatGPT use on learner autonomy remained significant [*B* = 0.193, β = 0.225, 95% CI (0.13, 0.24)], accounting for 65.24% of the total effect. The indirect effect transmitted through speaking anxiety was also significant [*B* = 0.103, β = 0.120, Boot *SE* = 0.013, 95% CI (0.07, 0.13)], representing 34.80% of the total effect. Figure 1 illustrates the complete mediation model with all standardized coefficients.

**Table 5:** Total, direct, and indirect effects of ChatGPT use on learner autonomy.

Effect type	*B*	β	Boot *SE*	Boot 95% CI	% of total effect
Total effect (X → Y)	0.296	0.344	0.027	[0.241, 0.350]	100.00%
Direct effect (X → Y)	0.193	0.224	0.028	[0.137, 0.248]	65.24%
Indirect effect via M (X → M → Y)	0.103	0.119	0.013	[0.078, 0.130]	34.80%

*B*, unstandardized effect; β, completely standardized effect; Boot *SE*, bootstrap standard error; CI, bias-corrected 95% confidence interval based on 5,000 bootstrap resamples. Indirect effect CI excludes zero, confirming significant partial mediation.

Critically, the bootstrap confidence interval for the indirect effect did not include zero, confirming that speaking anxiety functions as a significant partial mediator in the relationship between ChatGPT use and learner autonomy. These findings indicate that while ChatGPT use directly promotes learner autonomy, a meaningful portion of this relationship operates indirectly through the attenuation of English speaking anxiety.

## 4 Discussion

The present findings suggest that ChatGPT appears to function not merely as a linguistic tool but as an affective scaffold associated with reduced communicative inhibition, which in turn may be linked to greater independent engagement in self-directed language learning behaviors. It is important to acknowledge, however, that this interpretation rests on theoretical inference rather than direct measurement: the ChatGPT Usage Scale primarily captures writing and general academic support functions, and speaking-specific engagement was not assessed. Furthermore, any characterization of participants' ChatGPT engagement as habitual or sustained must be treated as a theoretical assumption, given that usage frequency and prior experience were not directly measured in the present study. Consequently, the relationship between ChatGPT use and speaking anxiety cannot be empirically verified from the present data alone, and should be interpreted as a theoretically plausible rather than directly evidenced pathway. Future research employing speaking-specific ChatGPT use measures would enable stronger claims regarding the affective scaffolding role of AI in oral language development.

A methodological consideration warranting explicit discussion concerns the use of the FLCSAS—a classroom-based speaking anxiety instrument—as the mediating variable in a model centered on ChatGPT use. This choice is theoretically justified by conceptualizing speaking anxiety not as a state experienced during AI interactions, but as a stable dispositional tendency reflecting chronic communicative inhibition in teacher- and peer-present contexts (Apple, 2013; Horwitz et al., 1986). It is theoretically assumed—though not empirically verified in the present study—that if ChatGPT engagement were sustained and low-stakes in nature, it could progressively restore perceived oral competence and reduce fear of negative evaluation, with these affective gains potentially generalizing from AI-mediated practice to classroom-based speaking dispositions (Bashori et al., 2021; Fauzi et al., 2025). The FLCSAS thus captures the downstream affective outcome theoretically targeted by ChatGPT use rather than in-session AI anxiety. Nevertheless, this generalization assumption was not directly tested, and future research employing paired measures of both AI-context and classroom-context anxiety would enable more rigorous construct-level verification of this mediating pathway.

The present findings align with and extend a growing body of empirical literature. The positive association between ChatGPT use and learner autonomy corroborates prior experimental and mixed-methods evidence demonstrating that AI-assisted learning produces meaningful autonomy gains relative to teacher-directed instruction (Lee et al., 2024; Nguyen and Hue, 2025; Xu et al., 2024). The negative relationship between ChatGPT use and speaking anxiety is consistent with studies documenting that AI-mediated communication reduces social-evaluative threat and affective filter activation (Bashori et al., 2021; Fauzi et al., 2025; Wardat and Akour, 2025). Furthermore, the inverse association between anxiety and autonomy replicates patterns observed in prior investigations linking affective barriers to reduced self-regulation (Liu et al., 2024; Sayed et al., 2024; Wei, 2023). Most importantly, the confirmed partial mediation extends earlier theoretical propositions by AlTwijri and Alghizzi (2024) and Crompton et al. (2024), providing the first empirical test of this sequential pathway within a single structural model. These findings should further be interpreted within the specific Egyptian public university context, characterized by large class sizes and limited authentic English exposure, which may amplify speaking anxiety and heighten the perceived utility of ChatGPT as an alternative practice space.

The findings carry theoretical and practical implications, though these should be interpreted cautiously given the acknowledged construct alignment and design limitations. Theoretically, the results are consistent with the integration of the Affective Filter Hypothesis and Self-Determination Theory as complementary explanatory frameworks for AI-mediated language learning, suggesting that anxiety reduction represents a psychologically plausible—though not directly evidenced—mechanism linking ChatGPT use to autonomous behavior. Practically, EFL instructors and curriculum designers may consider structuring ChatGPT-assisted speaking activities that deliberately target anxiety reduction—such as low-stakes AI conversation practice—as a potentially useful prerequisite to fostering greater learner autonomy; however, such recommendations remain tentative pending verification through experimental and longitudinal designs that directly measure speaking-specific ChatGPT engagement and its effects on both AI-context and classroom-context anxiety. Institutional administrators at Egyptian universities may benefit from developing guided AI integration policies that balance technological access with pedagogical scaffolding, ensuring that ChatGPT adoption has the potential to translate into authentic self-directed competence rather than passive tool dependence.

Several limitations should be acknowledged when interpreting the present findings. First, the cross-sectional design precludes causal inference. Second, self-report data introduce potential response bias. Third, the sample was drawn exclusively from three Egyptian public universities, limiting generalizability. Fourth, actual speaking proficiency and objective autonomy behaviors were not assessed. Fifth, and critically, participants' ChatGPT usage frequency, prior experience, and speaking-related engagement were not measured. This represents a meaningful conceptual gap: the ChatGPT Usage Scale operationalizes general and writing-oriented use, and the proposed relationship with speaking anxiety therefore relies on theoretical inference rather than construct-aligned measurement. Sixth, English proficiency level and demographic variables were not statistically controlled, limiting interpretability.

Future research should address the limitations identified above through several methodological extensions. Longitudinal designs incorporating repeated-measures assessments would enable stronger causal claims about the developmental trajectory of anxiety reduction and autonomy growth through sustained ChatGPT engagement. Experimental studies comparing structured vs. unguided ChatGPT-assisted speaking conditions would clarify the pedagogical mechanisms underlying the observed effects. Researchers should also expand sampling to diverse cultural and linguistic contexts to assess cross-national generalizability. Additionally, incorporating objective measures of oral proficiency alongside self-report indices would strengthen construct validity. Finally, investigating boundary conditions—such as the moderating roles of digital self-efficacy, language proficiency, and teacher support—would yield a more nuanced understanding of when and for whom ChatGPT use most effectively reduces anxiety and promotes autonomy.

## 5 Conclusion

This study provides preliminary correlational evidence consistent with the proposition that English speaking anxiety partially mediates the positive relationship between ChatGPT use and learner autonomy among EFL university students. By drawing on the Affective Filter Hypothesis and Self-Determination Theory within a unified mediation framework, the findings illuminate a theoretically coherent, though not causally verified, pathway through which AI-assisted language tools may be associated with lower affective barriers and greater independent learning tendencies. Given the cross-sectional design, self-report measures, and the construct boundary between the classroom-based FLCSAS and ChatGPT-centered use, these findings should be regarded as theoretically suggestive rather than conclusive. The results tentatively underscore the potential importance of anxiety-targeted AI integration strategies in EFL curricula and call for intentional pedagogical design that moves beyond mere technological access toward genuine learner empowerment. As generative AI continues to reshape language education, attending to its affective and motivational dimensions remains an indispensable priority for researchers and practitioners alike, and rigorous longitudinal and experimental research is needed to substantiate the causal claims implied by the present mediation model.

## Data Availability

The raw data supporting the conclusions of this article will be made available by the authors, without undue reservation.
